# Transplant Trial Watch

**DOI:** 10.3389/ti.2023.12423

**Published:** 2023-12-12

**Authors:** Simon R. Knight

**Affiliations:** ^1^ Centre for Evidence in Transplantation, Nuffield Department of Surgical Sciences, University of Oxford, Oxford, United Kingdom; ^2^ Oxford Transplant Centre, Churchill Hospital, Oxford, United Kingdom

**Keywords:** organ donation, randomised controlled trial, kidney transplantation, immunosuppresion, tacrolimus

To keep the transplantation community informed about recently published level 1 evidence in organ transplantation ESOT and the Centre for Evidence in Transplantation have developed the Transplant Trial Watch. The Transplant Trial Watch is a monthly overview of 10 new randomised controlled trials (RCTs) and systematic reviews. This page of Transplant International offers commentaries on methodological issues and clinical implications on two articles of particular interest from the CET Transplant Trial Watch monthly selection. For all high quality evidence in solid organ transplantation, visit the Transplant Library: www.transplantlibrary.com.

RANDOMISED CONTROLLED TRIAL 1 Randomized Intervention to Assess the Effectiveness of an Educational Video on Organ Donation Intent Among Hispanics in the New York Metropolitan Area.

*by Pekmezaris, R., et al. World Journal of Transplantation 2023; 13(4): 190–200*.

## Aims

The aim of this study was to evaluate whether an educational video was effective in improving organ donation intent among Hispanic New York residents.

## Interventions

Participants were randomised to either view a short educational video on organ donation prior to the survey or to view the same video following the survey.

## Participants

365 Hispanic New York City (NYC) residents.

## Outcomes

The main outcomes of interest were to assess the impact of the emotional video on willingness to donate, and to identify driving factors for organ donation.

## Follow-Up

N/A.

## CET Conclusion

This randomised study from New York recruited adult Hispanic residents and delivered an online survey to elicit their knowledge and views on organ donation. Participants were randomised to watch an emotive video on deceased donation either before answering the survey, or after. The authors found that participants who watched the video before answering the survey showed more willingness to register as a donor (OR 2.05) and greater awareness as to how to sign up. The study is well designed and interesting, demonstrating how simple information provision may impact donation decisions in diverse populations. It is worth noting that the study did not measure actual registrations, just intent, and future studies should look at impact on actual registration rates as a closer proxy to real-world benefit.

## Jadad Score

1.

## Data Analysis

Strict intention-to-treat analysis.

## Allocation Concealment

No.

## Trial Registration

N/A.

## Funding Source

Not reported.

RANDOMISED CONTROLLED TRIAL 2 Comparison of 2 Immunosuppression Minimization Strategies in Kidney Transplantation: The ALLEGRO Trial.

*by van den Born, J. C., et al. Transplantation 2023 [record in progress]*.

## Aims

The aim of this trial was to compare standard immunosuppression with two immunosuppression minimisation strategies in *de novo* kidney transplant recipients.

## Interventions

Participants were randomised to one of three groups: the early steroid withdrawal arm, the standard-dose tacrolimus arm, and the tacrolimus minimisation arm.

## Participants

295 *de novo* kidney transplant recipients.

## Outcomes

The primary outcome was kidney function at 24 months posttransplantation. The secondary outcomes were patient survival, treated rejection, kidney failure, discontinuation of study medication for more than 6 weeks, and treatment failure.

## Follow-Up

24 months.

## CET Conclusion

This multicentre trial from the Netherlands randomised *de novo* renal transplant recipients to one of three immunosuppression strategies—standard care, early steroid withdrawal or tacrolimus minimisation at 6 months. The study was designed to demonstrate non-inferiority in renal function at 24 months, and met the primary endpoint, with no difference seen between the three groups. There was a higher incidence of acute rejection in the early steroid withdrawal group, but no increase in DSA formation. In general, study design is good although unblinded, with centralised randomisation and intent-to-treat analysis. Withdrawal rate was around 25% in each arm at 24 months. Inclusion criteria are fairly broad for an immunosuppression minimisation study, allowing recipients up to 80 years of age, PRA up to 75% and first or second transplants. One notable exclusion criteria was for type 1 diabetic recipients; the authors do not provide a rationale for this.

## Jadad Score

3.

## Data Analysis

Strict intention-to-treat analysis.

## Allocation Concealment

Yes.

## Trial Registration

ClinicalTrials.gov—NCT01560572.

## Funding Source

Industry funded.

## Clinical Impact Summary

Whilst there have been relatively few randomised trials of novel immunosuppressant strategies in renal transplantation in recent years, there has been a lot of interest in modified protocols that aim to minimise the adverse effects of immunosuppressant agents. Most studies have focussed on minimising either corticosteroid use or calcineurin inhibitor exposure, as these have the potential to have greatest impact on long-term outcomes. Corticosteroid avoidance or minimisation appears to reduce the risk of metabolic complications (hypertension, high cholesterol and new onset diabetes) at the expense of slightly higher risk of early steroid sensitive acute rejection [1]. Calcineurin inhibitor withdrawal or tapering studies vary in their approach, with or without substitution with an mTOR inhibitor. Meta-analysis shows that CNI withdrawal or avoidance may increase the risk of acute rejection, but the reduced nephrotoxicity improves short-term graft survival and decreases the risk of hypertension [2]. However, heterogeneity in the published literature and a lack of long-term outcome reporting means that most centres still employ a long-term CNI strategy for renal recipients.

A recent, multicentre, randomised ALLEGRO study from the Netherlands attempts to address some of these issues [3]. In this open label study, *de novo* kidney transplant recipients were randomised to either standard immunosuppression (basiliximab, tacrolimus, MMF and steroids) or minimisation. Two different minimisation strategies were tested: early steroid withdrawal (at day 3) or late tacrolimus reduction (at month 6). Patients were followed for 24 months. The study followed a non-inferiority design, with a difference of 10 mL/min or less in eGFR at month 24 being defined as non-inferior. Both minimisation arms were shown to be non-inferior for the primary endpoint. As seen in previous studies (and meta-analyses) there was an increased incidence of acute rejection in the steroid withdrawal arm (but no increase in DSA formation), with a reduction in incidence of new onset diabetes and lower serum cholesterol. Unlike previous studies, there was no increase in rejection following late CNI minimisation.

The study design is robust, with central randomisation and use of an intent-to-treat analysis. Inclusion criteria for immunosuppression trials are often very restrictive and unrepresentative, so the authors should be commended for including a wide range of recipients up to 80 years of age, PRA of up to 75% and repeat transplants, improving real-world generalisability. Perhaps slightly oddly, the authors excluded recipients with pre-existing type 1 diabetes, but did not provide a rationale for this decision.

Whilst this is a large, well conducted study, the findings are not particularly novel and are in keeping with the existing literature. They do demonstrate, however, that late CNI reduction is safe and feasible. Longer-term follow-up of participants in the study would be useful to demonstrate the effects of a reduction in metabolic risk and/or nephrotoxicity on longer-term graft and patient survival.

### Clinical Impact

3/5.

## References

[1] Knight SR Morris PJ. Steroid Avoidance or Withdrawal After Renal Transplantation Increases the Risk of Acute Rejection But Decreases Cardiovascular Risk. A Meta-Analysis. Transplantation (2010) 89:1–14. 10.1097/TP.0b013e3181c518cc 20061913

[2] Karpe KM Talaulikar GS Walters GD. Calcineurin Inhibitor Withdrawal or Tapering for Kidney Transplant Recipients. Cochrane Database Syst Rev (2017) 7:CD006750. 10.1002/14651858.CD006750.pub2 28730648 PMC6483545

[3] van den Born JC Meziyerh S Vart P Bakker SJL Berger SP Florquin S Comparison of 2 Immunosuppression Minimization Strategies in Kidney Transplantation: The ALLEGRO Trial. Transplantation (2023). 10.1097/TP.0000000000004776 37650722

